# Chinese herbal medicine is associated with higher body weight reduction than liraglutide among the obese population: A real-world comparative cohort study

**DOI:** 10.3389/fphar.2022.978814

**Published:** 2022-09-09

**Authors:** Yu-Ning Liao, Hsing-Yu Chen, Ching-Wei Yang, Pai-Wei Lee, Chiu-Yi Hsu, Yu-Tung Huang, Tsung-Hsien Yang

**Affiliations:** ^1^ Division of Chinese Internal Medicine, Center for Traditional Chinese Medicine, Chang Gung Memorial Hospital, Taoyuan, Taiwan; ^2^ School of Traditional Chinese Medicine, College of Medicine, Chang Gung University, Taoyuan, Taiwan; ^3^ Graduate Institute of Clinical Medical Sciences, College of Medicine, Chang Gung University, Taoyuan, Taiwan; ^4^ Center for Big Data Analytics and Statistics, Chang Gung Memorial Hospital, Linkou Medical Center, Taoyuan, Taiwan; ^5^ Graduate Institute of Pharmacognosy, College of Pharmacy, Taipei Medical University, Taipei, Taiwan

**Keywords:** traditional Chinese medicine, obesity, overweight, weight loss, body mass index, liraglutide, weight control

## Abstract

**Introduction:** In Taiwan, many people receive Chinese herbal medicine (CHM) as an alternative choice to help control body weight. However, the clinical effectiveness of CHM on weight control has not been well studied, while potential risks and adverse effects are still unknown. The aim of our study is to find out a safe and efficient treatment model of CHM for weight control compared to liraglutide in a real-world setting.

**Methods:** we retrospectively analyzed obese subjects [body mass index (BMI)≧25 kg/m^2^] from Chang Gung Research Database (2013–2018). We evaluated the effect on body weight and BMI changes in obese groups receiving CHM or western medicine (WM, represented liraglutide) within 180 days. The proportion of subjects who achieved 5 and 10% weight reduction was calculated as well. Furthermore, the potential adverse events were analyzed during the study period. Overlap weighting was used to balance the baseline differences between CHM and WM groups.

**Results:** The full cohort comprised 1,360 participants: 701 in the CHM group and 659 in the WM group. At baseline, the CHM group was younger (42.75 ± 12.12 years old in CHM vs. 52.31 ± 11.7 years old in WM, *p*-value <0.001) and has more female subjects (77.6% in CHM vs. 53.0% in WM, *p*-value <0.001). On the other hand, CHM users had lower body weight (79.83 ± 15.66 kg vs. 84.68 ± 17.14 kg, *p*-value <0.001) and BMI (30.58 ± 5.20 vs. 32.84 ± 6.95, *p*-value <0.001). At day 180, CHM users lost more body weight (−4.5 ± 4.07 kg vs. −2.15 ± 4.05 kg, *p*-value <0.001) and higher reduction in BMI (−1.77 ± 1.73 vs. −0.9 ± 2.14, *p*-value <0.001). A total of 53.21% (n = 373) CHM users lost at least 5% of body weight (22.46% for WM users, *p*-value <0.001), and 18.97% (n = 132) lost at least 10% of body weight (4.55% for WM users, *p*-value <0.001). The benefit remained consistent with and without overlap weighting. For adverse events, 18 cases of hypertension occurred in 659 subjects in the WM group (2.7%) in comparison to 1 of 701 subjects in the CHM group (0.1%).

**Conclusion:** CHM led to clinically meaningful weight loss without serious adverse events in a real-world setting. Further clinical trials are warranted to validate this result.

## Introduction

Obesity, a worldwide epidemic issue, causes a global public health problem for both individuals and society (Stefan et al., 2013). According to “2013–2016 Nutrition and Health Survey in Taiwan”, the prevalence of being overweight and obesity (BMI≧24) in people above 19 years old is 45.4% (Chang et al., 2017). Obesity is correlated with the formation of hypertension, diabetes mellitus, hyperlipidemia, and other cardiovascular diseases. More seriously, being overweight and obese increases the risk of death from all causes, cardiovascular disease, cancer, or other diseases for both men and women in all age groups (Calle et al., 1999). For this reason, weight loss is a crucial pathway to health improvement for patients with obesity-associated risk factors and comorbidities (Ryan and Yockey, 2017). It would be useful for overweight patients who have been unsuccessful with lifestyle modification, diet, and exercise alone to combine their weight reduction methods with medications approved for chronic weight management. To date, as we know, the gradual progression of body weight loss is more likely to be maintained over a longer period of time (Mertens and Van Gaal 2000). Therefore, the novel anti-obesity strategy has focused on modest weight loss, defined as a weight loss of 5%–10% of an individual’s baseline weight, which has been demonstrated to reduce complications related to obesity and improve quality of life (Mertens and Van Gaal 2000; Warkentin et al., 2014; American College of Cardiology/American Heart Association Task Force on Practice Guidelines, Obesity Expert Panel, 2013, 2014).

The Food and Drug Administration (FDA) has approved five major weight loss medications available for weight management, which are orlistat, phentermine, phentermine/topiramate extended-release, naltrexone/bupropion sustained-release, and liraglutide (Müller et al., 2022). These anti-obesity drugs have been reported to have a statistically average mean weight loss of 3%–7% from the baseline in clinical trials (Srivastava and Apovian 2018; Müller et al., 2022). However, the safety or significant tolerability issues of the currently available anti-obesity medications arise in the long term use (Lee and Dixon 2017). Phentermine, a sympathomimetic agent, poses side effects, such as tachycardia, insomnia, constipation, and agitation. Commonly, phentermine is a short acting medication and short-term use is suggested since the suppression on appetite may wear off in several weeks (Apovian et al., 2015b). Orlistat, inhibiting pancreatic and gastric lipases, has adverse effects, including steatorrhea, oily spotting, and fecal incontinence, which is poorly tolerated (Apovian et al., 2015a). Topiramate, an anticonvulsant and also a centrally acting drug, has been reported with dose-related cognitive side effects, including psychomotor slowing, decreased concentration and attention, memory impairment, and an unexpected surge in suicidal thoughts (Shin and Gadde 2013). Liraglutide, a glucagon-like peptide-1 (GLP-1) agonist with, presently, the most promising weight-lowering effect, increases incidence of symptomatic gallstones and may elevate the risk of pancreatitis apart from gastrointestinal symptoms (Pi-Sunyer et al., 2015). Hence, safety, tolerability, and efficacy are three impartible core issues surrounding the commencement of weight-loss medications. As the long-term benefits are likely to be outweighed by the risks and costs of treatment, especially cardiovascular and mental health safety issues, new medications or even dietary supplements for obesity are still needed (Müller et al., 2022).

In accord with this approach, numerous complementary and alternative therapies have been used in the Eastern part of the globe for a long time, however, recently, these unconventional therapies are increasingly applied worldwide including Chinese herbal medicine (CHM) (Hasani-Ranjbar et al., 2009). CHM, being documented for thousands of years, has been applied as a form of health promotion and disease treatment throughout the world as a result of being a natural compound, which is regarded as a safer option than synthetic chemical agents (Ignjatovic et al., 2000). In Taiwan, there are many people receiving CHM to help control body weight. Nevertheless, the evidence base for therapeutic efficacy of CHM has not been widely verified by using international standards yet in spite of their extensive use in current clinical practices (Sui et al., 2012). Owing to little information about the efficacy of CHM on weight control, potential risks and adverse effects are confirmed. Thus, we investigated one of CHMs which is used as a therapeutic option to promote weight loss in our present clinical specialty. Ma-Xing-Gan-Shi-Tang (MXGST), a Chinese medicine formula, has been traditionally used to adjust the lung qi stagnation, clear the pathological heat, and reduce phlegm. In clinical practice, traditional Chinese medicine (TCM) doctors always judge the clinical symptoms and signs of individuals to adjust the prescription to achieve holistic effects. Based on TCM doctor’s clinical experience, MXGST may play a major role in the prescription to treat obesity, combating the overweight dilemma in several different ways in our clinical observations and speculations, such as improving the phlegm-dampness constitution by adjusting the lung qi stagnation. However, it lacks sufficient evidence-based studies or clinical trials to evaluate the promising weight-reducing effects of MXGST herbal formulation in present research.

The aim of this cohort study is to explore the safety and effectiveness of CHM treatment on weight control in comparison with liraglutide, and further attestation of its possible therapeutic values in this overwhelming pandemic of obesity.

## Materials and methods

### Preparation and composition of MXGST

MXGST used in our clinical practice is the dry powder derived from the water extract of a mixture of four botanical and mineral drugs, which contains *Ephedra sinica* Stapf, *Prunus armeniaca* L. var. *ansu* Maxim, *Glycyrrhiza uralensis* Fisch, and Gypsum fibrosum. Every 3 g of the water extract are derived from 20 g of the raw materials. The ratio of each botanical and mineral drugs is according to the authority of TCM in Taiwan and is presented in Table 1 (Ministry of Health and Welfare).

**TABLE 1:** The botanical and mineral drugs contained in Ma-Xing-Gan-Shi-Tang (every 3 g of the water extract are derived from 20 g of the raw materials).

The name of botanical and mineral drugs	Ratio (%w/W)
*Ephedra sinica* Stapf [Ephedraceae; Ephrdrae herba]	20.0
*Prunus armeniaca* L. var. *ansu* Maxim [Rosaceae; Armeniacae semen amarum]	20.0
*Glycyrrhiza uralensis* Fisch [Leguminosae; Glycyrrhizae radix et rhizoma]	10.0
Gypsum fibrosum	50.0

### Data source and study protocol

The Chang Gung Research Database (CGRD) was used as the data source of this study. The CGRD was composed of daily clinical practices, including all procedures and medications of outpatient, inpatient, and emergency visits in the Chang Gung Memorial Hospital (CGMH). The CGMHs provide CHM and Western medicine (WM) for patient care, comprising eight medical institutes with different hospital levels, and serve as the largest medical system in Taiwan (Ku et al., 2021). According to the statistics, CGMHs own 10,070 beds and admit more than 280,000 patients each year, supporting over 8,500,000 outpatient visits and 500,000 emergency department visits in 2015 (Shao et al., 2021). The huge amount of clinical data has made the CGRD become a great resource for clinical studies (Shao et al., 2021).

We retrieved the electronic medical records (EMR) from eight CGMHs to provide real-world evidence, which involved patient information demographics, clinical parameters, diagnostic information, prescription information, and other health care facility information (Shao et al., 2021). The selection period for treatment initiation was from 1 January 2013 to 31 December 2018, with baseline demographic data based on the 6 months period prior to initiation, including baseline weight, biochemical and physiological profiles, and co-morbidities. We selected MXGST as the CHM prescription to help weight-loss in the CHM group and chose liraglutide as the anti-obesity medication in the WM group. Index date was the date when MXGST or liraglutide treatment was initiated during the selection period. The end date of the follow-up was the last return appointment after a 6 months therapeutic period. Patients were evaluated every month until day 180. All patients received standardized counseling on CHM or WM weight-loss prescription by a clinical physician on an approximately monthly basis with their weight reduction as well as any documentation of adverse events. To reveal the trend of body weight reduction in the 6-months follow-up duration, the subjects’ body weight changes at day 30, 60, 90, 120, 150, and 180 after the start of treatment were retrieved for this study. In this observational real-world study, body weight was compared retrospectively with the measured value at the start of treatment. The study design and protocol were reviewed and approved by the Institutional Review Board of the Chang Gung Memorial Foundation (IRB No: 201801526B0). The need for informed consent was waived because the identification number of each subject was well-encrypted and therefore it was impossible to recognize the real identity of each subject.

### Study population and covariates

The International Classification of Diseases, 10th Revision, Clinical Modification (ICD-10-CM) and Ninth Revision, Clinical Modification (ICD-9-CM) were used to determine obese population. We included patients who were properly diagnosed with obesity or metabolic syndrome between 2013/01 and 2018/12 from the CGRD (ICD-9-CM codes: 278.0, 278.00, 278.01, 278.03, 649.10–14, 793.91, V85.30–39, V85.41–45, V85.54, and ICD-10-CM codes: E65, E668, E669). The highest body weight around 1 month before or after the index date and the following body weight check-up dates were recognized as the body weight of each time point. Subjects were included in this study if they fulfilled all of the following criteria: 1) subjects were ≥18 years old and ≤75 years old at the index date; 2) they had a body mass index (BMI; the weight in kilograms divided by the square of the height in meters) ≥25 kg/m^2^ with at least one weight-related disease such as prediabetes, diabetes mellitus (DM), hypertension (HTN), dyslipidemia, or hepatic steatosis during the 360 days before the index date; 3) they had at least one prescription for MXGST or liraglutide at the discretion of the physician; and 4) they had at least one reported baseline body weight measurement within 1 month before the index date; and those who continued to visit the clinic after 1–2 months were treated with MXGST or liraglutide after the index date. The exclusion criteria were listed as follows: 1) missing weight or height record from EMR at baseline and around the end date of follow-up; and 2) taking other anti-obesity medications or a history of bariatric surgery. We collected data of people who met the inclusion criteria as afore mentioned at first visit to outpatient department from the CGRD, including the visit date, age, gender, height, weight, blood pressure, heart rate, underlying comorbidities, information about the use of CHM and WM, laboratory data, and examinations.

### Outcome assessment

Our retrospective study assessed the weight-loss efficacy of MXGST in the CHM group and liraglutide in the WM group as well as anti-obesity therapy-related adverse effects in this cohort according to the 6 months of treatment of compliance with these prescriptions. The primary outcome was the body weight change from baseline to the 180th day. The secondary outcome was the proportion of subjects who lost at least 5% of their baseline body weight, and the proportion of subjects who lost more than 10% of their baseline body weight. To compute BMI, we used the average value to impute subjects’ body height if not recorded; however, to disclose the real-world status, the missing value of body weight on 30-, 60-, 90-, 120- and 150-days body weight was not imputed in the final analysis. Besides, we also evaluated whether there are potential adverse effects, such as cerebrovascular disease and cardiovascular system impairment. We scrutinized adverse events that occurred during the 6 months therapeutic period, including the medical records of emergency room or hospitalization with onset on or after the first day of treatment and no later than 30 days after the last day of treatment.

### Statistical analysis

For baseline demographic features, descriptive statistics with X^2^ statistics and independent t-tests were used to examine the differences between CHM and WM users. Moreover, independent t-tests were used to examine the weight changes between two groups, while X^2^ statistics were used to examine the differences of the proportion of 5%- and 10%-weight reduction and adverse events between two groups at the end of study. Also, overlap weighting was used to minimize the potential confounding bias caused by the different baseline features of CHM and WM users. Overlap weighting is a propensity score (PS)-based statistical method widely used in observational studies to mimic randomized clinical trials, especially when considering case imbalance or potential biases caused by prominently different initial status (Li, Thomas, and Li 2019; Thomas, Li, and Pencina 2020). In this study, age, gender, Charlson Comorbidity Index (CCI), mean arterial pressure (MAP), and body weight were used to generate the PS. PS was assigned as the weight of WM users while 1-PS was assigned to CHM users before effect estimation. On the other hand, we also conducted sensitivity tests by using other PS-related models, including 1:1 propensity score matching and Inverse probability of treatment weighting (IPTW) to examine the effect of the CHM treatment. All statistical calculations were carried out by SAS and results with a *p*-value <0.05 was considered to be statistically significant.

## Results

### Baseline characteristics of study patients

**FIGURE 1:**
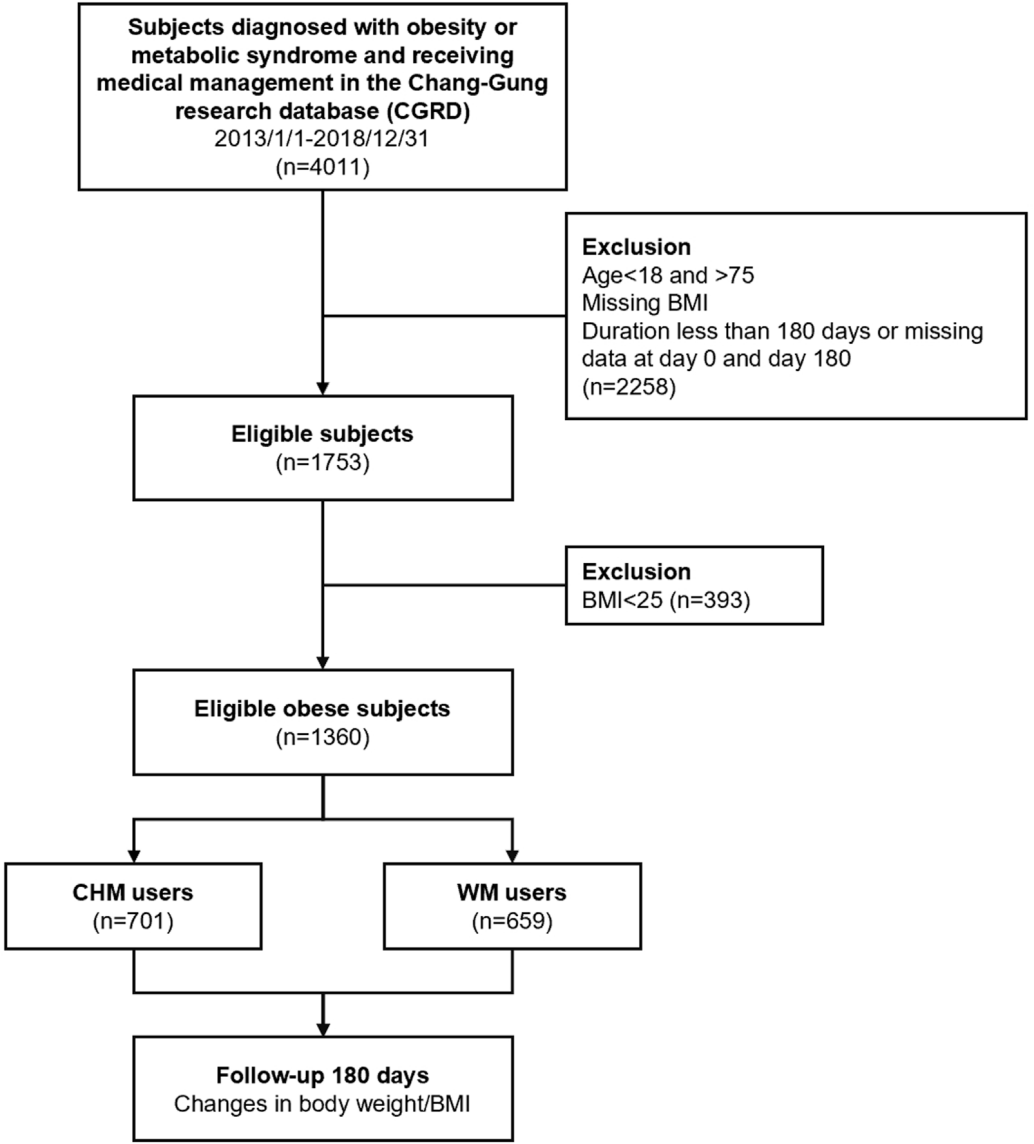
A flowchart of the collection of subjects from the CGMH hospital outpatient database from 2013 to 2018 in Taiwan.

During 2013 and 2018, there were 701 CHM users and 659 WM users entering the final analysis stage who completed 180 days of treatment (Figure 1). The baseline clinical characteristics of subjects with CHM and WM treatment are shown in Table 2. Overall, CHM users tended to be younger than WM users, 42.75 and 52.31 years old, respectively (*p*-value <0.001), and a higher proportion of female subjects was found among CHM users, 77.6 vs. 53.0% WM users, respectively (*p*-value <0.001). Additionally, a higher proportion of WM users had hypertension, dyslipidemia, and diabetes mellitus (DM) (CHM vs. WM users, 11.7 vs. 61.8% for hypertension, *p*-value <0.001; 9.1 vs. 67.1% for dyslipidemia, *p*-value <0.001; and 5.6 vs. 77.4% for DM, respectively, *p*-value <0.001), and a higher CCI (0.30 for CHM vs. 2.33 for WM users, respectively, *p*-value <0.001). As to biochemical and physiological profiles, there were prominent differences in MAP, body weight, BMI, and lipid profiles, which were higher in the WM group (all *p*-values <0.05). However, WM users seemed to have a higher HbA1C and fasting glucose level than CHM users, 9.82 vs. 6.51% for HbA1C and 213.49 ± 73.45 mg/dl vs. 106.1 ± 33.82 mg/dl for fasting glucose level, respectively (*p*-value <0.001). The use of overlap weighting properly eliminated the differences in baseline features among CHM and WM users in age, gender, CCI index, MAP, and body weight covariates (Table 3).

**TABLE 2:** Baseline demographic features of study subjects.

	CHM user	WM user	*p*-value
	(n = 701)	(n = 659)	
Demographics			
Age—years	42.75 ± 12.12	52.31 ± 11.7	<0.001
Gender—no. (%)			
Male	157 (22.4)	310 (47.0)	<0.001
Female	544 (77.6)	349 (53.0)	<0.001
Smoker	5 (0.71)	73 (11.08)	<0.001
Alcohol drinker	3 (0.43)	39 (5.92)	<0.001
Betel nut user	0 (0.00)	17 (2.58)	<0.001
Comorbidities (%)			
Hypertension	82 (11.7)	407 (61.8)	<0.001
Dyslipidemia	64 (9.1)	442 (67.1)	<0.001
Ischaemic heart diseases	6 (0.8)	81 (12.3)	<0.001
Diabetes mellitus	39 (5.6)	510 (77.4)	<0.001
Chronic hepatitis	16 (2.3)	83 (12.6)	<0.001
Fatty liver	4 (0.6)	9 (1.4)	0.22
Nonalcoholic steatohepatitis	1 (0.1)	12 (1.8)	0.004
CCI	0.3 ± 0.7	2.33 ± 1.59	<0.001
Biochemical and physiological profiles (mean ± SD)			
MAP—mmHg	131.7 ± 15.1	128.8 ± 15.28	0.001
Weight—kg	79.38 ± 15.66	84.68 ± 17.14	<0.001
BMI—kg/m^2^	30.58 ± 5.2	32.84 ± 6.95	<0.001
AST—mg/dL	32.18 ± 15.94	49.44 ± 79.4	0.003
ALT—mg/dL	35.84 ± 32.2	48.46 ± 46.98	<0.001
BUN—mg/dL	13.1 ± 4.79	25.72 ± 19.02	<0.001
Creatinine—mg/dL	0.71 ± 0.2	1.10 ± 0.98	<0.001
HbA1C—%	6.51 ± 1.25	9.82 ± 1.7	<0.001
Fasting glucose—mg/dL	106.1 ± 33.82	213.49 ± 73.45	<0.001
Lipid profile (mean ± SD)			
Total cholesterol—mg/dL	196.31 ± 29.78	186.12 ± 44.45	0.004
Triglyceride—mg/dL	152.83 ± 76.84	230.06 ± 222.2	<0.001
LDL cholesterol—mg/dL	121.65 ± 28.86	104.83 ± 32.63	<0.001
HDL cholesterol—mg/dL	45.34 ± 10.01	40.12 ± 10.1	<0.001

*Abbreviations: CCI, charlson comorbidity index.

*Continuous covariates are presented as the median (interquartile range, IQR) while categorical covariates are presented as a number (percentage).

*Body mass index (BMI) is the weight in kilograms divided by the square of the height in meters.

*The *p*-value was calculated by means of Fisher’s exact test, Pearson’s chi-squared test, and Student’s t-test on the basis of the number of participants.

**TABLE 3:** Baseline characteristics of subjects were balanced by using overlap weighting.

	Without overlap weighting			With overlap weighting		
	CHM user	WM user	SMD	CHM user	WM user	SMD
Demographics						
Age—years	42.75 ± 12.12	52.31 ± 11.7	0.803	47.84 ± 5.53	47.84 ± 5.54	0.000
Gender—no. (%)			0.535			0.000
Male	157 (22.4)	310 (47)		33.3	33.3	
Female	544 (77.6)	349 (53)		66.8	66.8	
Comorbidities						
CCI	0.3 ± 0.7	2.33 ± 1.59	1.653	0.97 ± 0.51	0.97 ± 0.31	0.000
Biochemical and physiological profiles						
MAP—mmHg	131.7 ± 15.1	128.8 ± 15.28	0.191	132.1 ± 6.81	132.1 ± 7.68	0.000
Weight—kg	79.38 ± 15.66	84.68 ± 17.14	0.323	82.27 ± 16.95	82.77 ± 16.28	0.000

*Abbreviations: CCI, charlson comorbidity index.

*Continuous covariates are presented as the median (interquartile range, IQR) while categorical covariates are presented as a number (percentage).

*The balance of covariates using the standardized mean difference (SMD).

*An SMD ≤0.1 indicates a negligible difference in potential confounders between the two study groups.

*Weighted covariates were age, gender, CCI, index, and body weight.

### Body weight reduction

**FIGURE 2:**
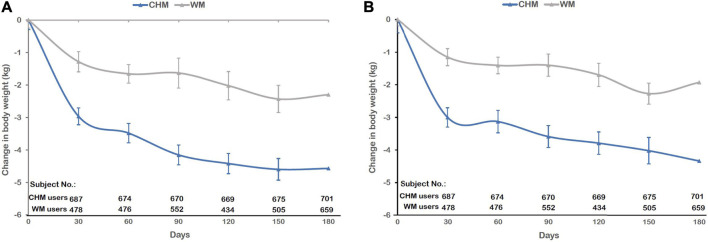
Changes in body weight after 180 days of CHM and WM treatment. **(A)**Without overlap weighting and **(B)** with overlap weighting.

After 180 days, the use of CHM led to a significantly greater reduction in body weight than WM. CHM users had lost 4.57 ± 4.51 kg of their body weight, whereas WM users had lost 2.3 ± 5.48 kg of their body weight at the end of study (Table 4). Weight loss from the baseline during the study is shown in Figure 2. The trend of estimated mean weight loss in this cohort study was significantly greater with CHM than with WM from stem to stern. A greater reduction in the body weight of CHM users than WM users was observed (at day 30, −2.9 ± 3.36 kg vs. −1.28 ± 2.71 kg; at day 60, −3.48 ± 3.45 kg vs. −1.66 ± 3.45 kg; at day 90, −4.2 ± 3.82 kg vs. −1.63 ± 3.44 kg; at day 120, −4.46 ± 3.82 kg vs. −1.85 ± 3.61 kg; at day 150, −4.64 ± 3.98 kg vs. −2.28 ± 3.96 kg; and at day 180, −4.5 ± 4.07 kg vs. −2.15 ± 4.05 kg) (Table 5). After overlap weighting, the data also revealed the significant weight loss difference between CHM users and WM users (Table 5). Body weight loss was the most significant within the first month and gradually plateaued since day 150 in the CHM group as well as in the WM group with a smaller slope (Figure 2).

**TABLE 4:** Changes in primary end point between the baseline and at day 180.

	Without overlap weighting			With overlap weighting		
	CHM user	WM user	*p*-value	CHM user	WM user	*p*-value
Changes in Body Weight	−4.57 ± 4.51	−2.3 ± 5.48	<0.001	−4.34 ± 2.54	−1.93 ± 2.02	<0.001
Kilograms of body weight						
% of body weight	−5.74	−2.62	<0.001	−5.15	−2.29	<0.001
Loss of >5% body weight—no. (%)	373 (53.21)	148 (22.46)	<0.001	45.9	19.51	<0.001
Loss of >10% body weight—no. (%)	132 (18.97)	30 (4.55)	<0.001	15.64	4.5	0.001
Changes in BMI	−1.77 ± 1.73	−0.9 ± 2.14	<0.001	−1.67 ± 0.98	−0.77 ± 0.94	<0.001

*The *p*-value was calculated by Pearson’s chi-squared test and Student’s t-test.

**TABLE 5:** Body weight reduction at day 30, 60, 90, 120, 150 and 180 after the start of treatment.

Day	CHM user		WM user			Overlap weighting		
	Subjects	△Body weight	Subjects	△Body weight		CHM user	WM user	
	(n)	(kg)	(n)	(kg)	*p*-value	△Body weight (kg)	△Body weight (kg)	*p*-value
30	687	−2.9 ± 3.36	478	−1.28 ± 2.71	<0.0001	−2.76 ± 1.81	−1.15 ± 1.03	<0.0001
60	674	−3.48 ± 3.45	476	−1.66 ± 3.45	<0.0001	−3.13 ± 1.81	−1.41 ± 1.4	<0.0001
90	670	−4.2 ± 3.82	552	−1.63 ± 3.44	<0.0001	−3.73 ± 1.88	−1.4 ± 1.46	<0.0001
120	669	−4.46 ± 3.82	434	−1.85 ± 3.61	<0.0001	−3.94 ± 1.82	−1.68 ± 1.67	<0.0001
150	675	−4.64 ± 3.98	505	−2.28 ± 3.96	<0.0001	−4.17 ± 1.87	−2.26 ± 1.89	<0.0001
180	701	−4.5 ± 4.07	659	−2.15 ± 4.05	<0.0001	−4.11 ± 1.93	−1.92 ± 1.97	<0.0001

**FIGURE 3:**
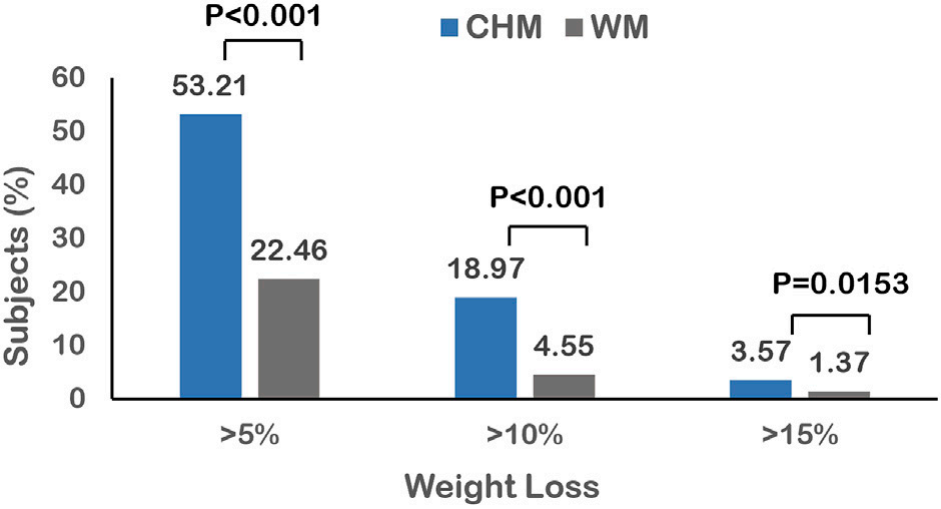
The differences in proportion of obese subjects achieving 5, 10, and 15% at day 180 between CHM and WM users.

After the 6-months treatment, 53.21% (n = 373) of the subjects in the CHM group lost more than 5% weight from baseline, which was significantly more than that in the WM group (22.46%, n = 148, *p*-value <0.001). The proportion of people losing more than 10% of their baseline weight was greater with CHM treatment (18.97%, n = 132) than with WM treatment (4.55%, n = 30, *p*-value <0.001). Furthermore, more individuals (3.57%, n = 25) treated with CHM lost more than 15% of baseline weight than those treated with WM (1.37%, n = 9, *p*-value <0.001) (Figure 3).

**FIGURE 4:**
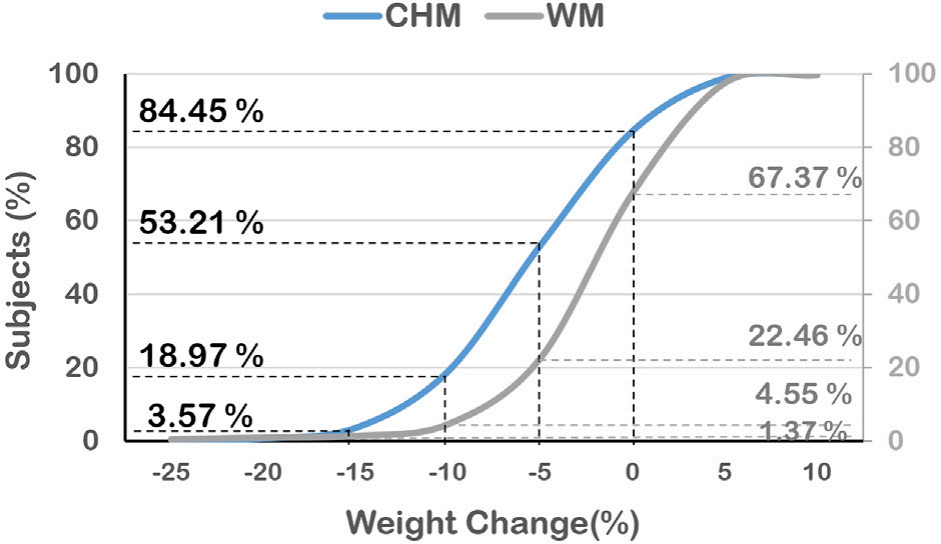
The differences in distribution of the percentage of reduced body weight among obese subjects at day 180 between CHM and WM users.

Overall, approximately 84.45% of the subjects in the CHM group vs. 67.37% of the subjects in the WM group lost weight after treatment in this trial. The majority of subjects had lost more than 5%–10% of body weight in the CHM group and the maximum weight loss didn’t exceed 20% of body weight after 180 days of treatment. Instead of losing weight, weight gain wasn’t beyond 5% of body weight in both groups (Figure 4). The CHM group also had a greater reduction than the WM group in mean BMI (1.77 ± 1.73 vs. 0.9 ± 2.14, *p*-value<0.001) (Table 4). Adjusted with overlap weighting, the trend of body weight loss in both groups was similar to the original data (Table 4 and Figure 2). Several sensitivity analyses confirmed the superiority of CHM over WM with respect to the primary end point as well. In the IPTW model, CHM users lost more body weight than WM users on treatment course Day 30, 60, 90, 120, 150, and 180 (*p*-value <0.0001). In the PSM model, CHM users also lost more body weight than WM users on treatment course Day 30, 60, 90, 120, 150, and 180 (*p*-value <0.05). These two models showed the consistent result that CHM users had significant weight loss than WM users during the whole treatment course (Supplementary Tables S1, S2 in the Supplementary Appendix SA1).

The subgroups analyses reveal that both in the groups older or younger than the median age, 48 years old, CHM users lost more body weight than WM users on treatment course Day 30, 60, 90, 120, 150, and 180 (*p*-value <0.0001). As to comorbidity subgroups, in the subgroup with diabetes mellitus, there is no significant difference in body weight loss between CHM users and WM users. In the subgroup without diabetes mellitus, CHM users lost more body weight than WM users on treatment course Day 30, 60, 90, 120, 150, and 180 (*p*-value <0.01). In the subgroup with hypertension, CHM users lost more body weight than WM users on treatment course Day 30, 60, 90, 120, 150, and 180 (*p*-value <0.05). In the subgroup without hypertension, CHM users lost more body weight than WM users on treatment course Day 30, 60, 90, 120, 150, and 180 (*p*-value <0.001). In addition, among 25≦BMI<30, 30≦BMI<35 and 35≦BMI subgroups, CHM users also lost more body weight than WM users on treatment course Day 30, 60, 90, 120, 150, and 180 (*p*-value <0.001) (Supplementary Table S3 in the Supplementary Appendix SA1).

### Side effects and adverse events

At the end of study, 18 cases of hypertension occurred in 659 patients in the WM group (2.7%) in comparison to 1 case in 701 patients in the CHM group (0.1%). Furthermore, the incidence of ischaemic heart disease events was higher in WM users than in CHM users (2 cases occurred in 659 subjects in the WM group (0.3%) in comparison to 0 cases in 701 subjects in the CHM group, *p*-value = 0.055), however, there was no statistical difference observed (Table 6). There were also no reported cases of cerebrovascular disease or brain disease occurring in both group treatments. No adverse effects with respect to hypertensive encephalopathy were observed in both groups by the end of the trial (Table 6). The diagnosis codes used in the study is presented in Supplementary Table S4 in the Supplementary Appendix SA1.

**TABLE 6:** Adverse events during the treatment period.

	CHM user	WM user	*p*-value
	(n = 701)	(n = 659)	
The Incidence of Adverse Events—no. (%)			
Hypertension	1 (0.1)	18 (2.7)	<0.001
Ischaemic heart disease	0	2 (0.3)	0.055
Cerebrovascular disease	0	0	—
Brain disease	0	0	—
Hypertensive encephalopathy	0	0	—

*The *p*-value was calculated by Pearson’s chi-squared test and Student’s t-test.

## Discussion

### Overall findings

The main findings of this study are as follows: First, CHM users led to a significantly greater reduction in body weight (primary end point) than WM users. The mean change in body weight in the CHM group was −5.74% (−4.57 ± 4.51 kg), which was shown to be superior to the WM group with respect to the primary end point after 180 days of treatment. The treatment effect was similar across the baseline BMI categories. Second, subjects with the CHM only reported an adverse event once, which was related to hypertension. Unlike currently prescribed anti-obesity drugs, the use of the CHM had the distinct advantage of significantly reducing adverse events compared with WM use.

### The clinical significance of CHM for weight loss

In terms of results, the CHM group had greater weight loss than the WM group and it seems that the CHM could afford substantial help for achieving modest weight loss, a weight loss of 5–10% from the baseline weight, that has been verified to decrease comorbidities associated with obesity and promote better quality of life in previous studies (Mertens and Van Gaal 2000; Warkentin et al., 2014). As far as we know, overweight or obesity is associated with higher risk for mortality and cardiometabolic diseases, and many studies have demonstrated that a modest 5% of weight loss can produce clinically meaningful improvement in various risk factors, comorbid diseases, and mortality that are associated with obesity (Ryan and Yockey, 2017). It has been reported that 5% of weight loss is capable of decreasing the plasma concentration of biomarkers related to cardiometabolic diseases, improving liver and adipose tissue insulin sensitivity, and lowering fasting glucose and hemoglobin A1c. (Magkos et al., 2016). As to risk factors of cardiovascular disease (CVD), 2%–5% of weight loss can improve hypertriglyceridemia and systolic blood pressure, 5% of weight loss can decrease intra-hepatic triglyceride by 13%, and 5%–10% of weight loss can improve diastolic blood pressure and high-density lipoprotein cholesterol level (Magkos et al., 2016). Besides, obesity is one of important risk factors of knee osteoarthritis (Raud et al., 2020). Patients who achieved more than 5% of weight loss would produce significant improvements in knee functionality, speed, walking distance, and pain (Messier et al., 2004). Research also indicated that each pound of weight loss would result in a 4-fold reduction in the load exerted on the knee per step during daily activities (Messier et al., 2005). Moreover, even a minimal weight loss of only 2%–5% of total body weight can improve ovulatory function by reducing ovarian volume and micro-follicle number, and is more likely to result in spontaneous pregnancy (Kiddy et al., 1992; Crosignani et al., 2003). Our cohort study demonstrated that more than half of CHM users could lose >5% of baseline body, although the therapeutic window is only limited to 180 days, which may be too short to confirm the influence of Chinese herbal medicine on diseases for the long term. This result inspired us that targeted health outcome goals may be reached by an individual with more than 5%–10% weight loss. The anti-obesity CHM has the benefits of not only achieving a weight loss goal, but also reaching targeted health outcome goals. What is meaningful about our study is that patients don’t have to reach the level of BMI <25 kg/m^2^ in all instances, and they can be healthier at any weight as long as they have a moderate weight loss.

When it comes to adverse events in taking medications, the herbal formulae used in the CHM group showed better tolerability than in the WM group (). The reasons to why CHM group had better weight-loss effects than the WM group in our cohort study are as following: First, CHM users had a larger proportion of female subjects (77.6 vs. 53.0% among WM users, *p*-value <0.001) and their average age was younger than WM users (42.75 years old for CHM vs. 52.31 years old for WM users, *p*-value <0.001). Previous studies reported that women and younger participants more easily achieved higher acceptable weight loss percentages due to powerful motivation concerning the increasing social desirability of weight loss which is associated with body image dissatisfaction and awareness of illness prevention (Czeglédi 2017; Nguyen et al., 2021). Second, WM users consisted of a greater proportion of seniors who may face challenges complicated by the progressive loss of skeletal muscle and accumulation of excess adipose tissue, which has been commonly referred to as sarcopenic obesity (Coker and Wolfe 2018). Given the lower level of aerobic fitness, a higher proportion of lean body mass loss, and a progressively decreasing metabolic rate, sarcopenic obesity may become a part of clinical conundrum in older individuals with obesity in the WM group (Coker and Wolfe 2018). Third, a higher proportion of WM users had hypertension, dyslipidemia, and DM. Chronic health problems like hypertension, DM, and endocrine problems may lead to a lower metabolic rate and make it harder to lose weight (Ganguly et al., 2018). The complexity of conditions in patients with more comorbidities may lead to less body weight loss in participants with metabolic syndrome than in those without such comorbidities (Zhou et al., 2012).

As to weight reduction concepts in TCM, the imbalance of the physiological state in energy (yang) deficiency, materials (yin) deficiency, or phlegm-stasis constitution may cause a high tendency of obesity (Hou et al., 2021). The TCM doctors’ prescriptions should adhere to the philosophy of CHM emphasizing on “personalized therapy” and TCM doctors would judge the clinical symptoms and signs of individuals to adjust CHM treatments for different people, being effective in reducing the side effects promptly during treatment (Sui et al., 2012). It is in contrast to Western medical doctors who conform to the uniform therapeutic guidelines and prescribe similar medications to patients with the same clinical diagnosis. This difference in therapeutic approach may partly explain the higher efficacy and lower incidence of adverse effects in the CHM group than in the WM group in the real-world (Sui et al., 2012). Given the health burden of obesity and metabolic syndrome, complementary use of the CHM is a possible option to address this unmet therapeutic aspect. Ma-Xing-Gan-Shi-Tang (MXGST) is an oriental herbal formula that has traditionally been used in patients with phlegmatic asthma and productive cough. MXGST is composed of *Ephedra sinica* Stapf (Ma-huang), *Glycyrrhiza uralensis* Fisch (Gan-cao), Gypsum fibrosum (Shi-gao), and *Prunus armeniaca* L. var. *ansu* Maxim (Xing-ren). The exact anti-obesity mechanisms of MXGST have not been validated yet, so we review experimental and clinical studies to infer the possible mechanisms. To our knowledge, MXGST might solve the body fat dilemma in several ways. Ma-huang has been applied to treat asthma, common cold, edema, arthralgia, and other symptoms in Asia for over 5,000 years (Ding 2009). Ma-huang is enriched with ephedrine-type alkaloids, of which ephedrine is the most abundant and active isomer. Ephedrine is a potential slimming drug that mediates thermogenic effects, primarily by the enhancement of sympathetic neuronal release of norepinephrine (NE) and epinephrine in both humans and laboratory animals (Bukowiecki, Jahjah, and Follea 1982; Dulloo, Seydoux, and Girardier 1992; Dulloo 1993). Given that brown adipocyte is an important site of catecholamine-induced thermogenesis in homeotherms (Bukowiecki, Jahjah, and Follea 1982), previous *in vitro* research revealed that ephedrine mimics the calorigenic action of norepinephrine by stimulating brown adipocyte respiration directly via beta-adrenoceptors (Ramsey et al., 1998). Gan-cao, has long been used as a traditional herbal medicine for stomach-invigorate and coordinating the drug actions of a prescription. Previous animal research had demonstrated that the extract of licorice root has beneficial influences on preventing atherosclerotic lesion development which is associated with inhibition of low-density lipoprotein (LDL) oxidation in hypercholesterolemic rats (El-Beshbishy et al., 2006). Some studies further provided evidence on anti-obesity properties of licorice root which can lower total cholesterol (TC) and low-density lipoprotein (LDL) in patients with hypercholesterolemia, being effective in reducing abdominal fat deposition and improving lipid profiles (Fuhrman et al., 2002; Bell, Canale, and Bloomer 2011; Mirtaheri et al., 2015). It seems that supplementation with Gan-cao may efficiently improve the lipid profile in overweight and obese subjects. Shi-gao, which is mainly composed of CaSO4, has been used as a treatment for reducing fevers and alleviating thirst in various TCM (Yuan et al., 2002; Ikarashi et al., 2013). It has been reported that the Shi-gao plays an important role in promoting urination and draining dampness in Chinese indigenous medicine and pharmacology. More recently, previous research proved that a combined administration of Ma-huang and Shi-gao would increase urine excretion (Huo et al., 2015). We conclude that Shi-gao may be a useful herbal medicine for changing the distribution of body fluid and increasing urine excretion with the highest potential to eliminate edema. Xing-ren, of which Amygdalin is a significant component (Jaswal et al., 2018), and is rich in oil, can promote intestinal peristalsis and prevent constipation. It has been used as a traditional herbal medicine for relieving constipation (Aritomi, Kumori, and Kawasaki 1985; Du et al., 2005; Chang et al., 2006; Erdogan-Orhan and Kartal 2011). We suggest that administering Xing-ren to improve bowel movements may be helpful for cleaning the accumulation of waste and toxins in the body. Based on our knowledge, it seems that the anti-obesity mechanisms of MXGST are remarkably different from liraglutide, which induces weight loss by delaying gastric emptying as well as suppressing appetite and energy intake (van Can et al., 2014; Knudsen and Lau 2019). In brief, MXGST has the potential for promoting metabolism, moistening intestines to relieve constipation, and regulating blood viscosity, making it has an obvious curative effect for losing weight. Thus, our retrospective cohort study demonstrates the efficacy and safety of MXGST in the management of obesity. Our results suggest that the MXGST treatment has a favorable benefit-risk ratio in obese groups. Careful observations and sophisticated surveillance are needed to investigate further the safety and long-term effects of this medication.

### The differences between real-world settings and RCTs

Our cohort study had several differences compared with other randomized controlled trial (RCT) studies. It may reveal that the weight loss effect of liraglutide in our real-world study in Asian populations was significantly less than that reported in RCT studies in Western populations. Liraglutide, a glucagonlike peptide-1 analogue, has been assessed by several randomized controlled trials indicating that it has anti-obesity effects possessing beneficial effects on glycemic control and could lower the risk of cardiovascular death in obese individuals (Howell, Wright, and Clements 2019). In our study, there are 22.46% (n = 148) and 4.55% (n = 50) of WM users who showed ≥5 and ≥10% body weight reduction, respectively, and the mean change in body weight of WM users was −2.62% (−2.3 ± 5.48 kg) at the end of the 180-days treatment. Previous research reported that patients who are overweight or obese in liraglutide group, the mean weight loss with liraglutide (administered subcutaneously in daily doses: 1.2–3.0 mg daily) was 4.8–7.2 kg in a 20-weeks randomized trial (Astrup et al., 2009). Additionally, in a 56-weeks trial, patients receiving once-daily subcutaneous injections of liraglutide, 3.0 mg, had lost a mean of 8.4 ± 7.3 kg of body weight, and approximately 63.2% of liraglutide users had lost at least 5% of their initial body weight (Pi-Sunyer et al., 2015). Another 56-weeks liraglutide 3.0 mg randomized trial had demonstrated that 81.4% of participants had maintained above 5% weight loss, and they had lost a mean of 6.2% of their initial body weight from randomization to the 56th week (Wadden et al., 2013). It seems that the body weight reduction effect of liraglutide observed in our study was slightly lower than that reported in RCTs. The reasons we presumed are as following: First, the baseline BMI of the subjects enrolled in our cohort study was 32.84 ± 6.95 kg/m^2^, which was lower than the average BMI >35 kg/m^2^ reported in previous studies of mostly Western populations (Astrup et al., 2012; Wadden et al., 2013; Davies et al., 2015; Pi-Sunyer et al., 2015). The mean body weight of our subjects showed a similar situation, which was much lower than the average body weight in the majority of previous studies of Western populations (84.68 ± 17.14 kg in our WM group vs. a body weight >105 kg in previous Western trials) (Pi-Sunyer et al., 2015; Davies et al., 2015; Wadden et al., 2013; le Roux et al., 2017). A previous study had found that absolute and relative reductions in body weight were dependent on the baseline BMI, which means that those populations with higher baseline BMI would lose more relative body weight than those with a lower baseline BMI (Ponzani 2013; Chitnis et al., 2014). It is consistent with the findings of our study that Asian ethnic groups, which do not usually have a very high BMI, are associated with less weight loss with liraglutide treatment. Second, liraglutide has been prescribed as a therapeutic agent to provide glycemic control for type 2 diabetes mellitus (T2DM), which would not be applied with the maximal dose daily for weight control purposes (Nauck et al., 2009). The retrospective nature of our study showed a higher proportion of T2DM individuals in the WM group under submaximal dosage of liraglutide for glycemic control. It may infer that the maximal medication benefits of reducing appetite and energy intake were not achieved in the WM group. Additionally, there were a wider range of subjects with T2DM comorbidity in the WM group. Some studies found that the complexity of conditions in patients with T2DM may lead to less body weight loss in participants with T2DM than in those without T2DM (Apovian et al., 2015a; Thomas et al., 2020). Third, chances are high that subjects’ motivation would de-escalate as time goes by, and it probably would result in low adherence after 180 days of treatment. In view of considerable variability in the general population and fluctuating compliance to anti-obesity treatment, the results of RCTs cannot always reflect the responses in real-world clinical practices (Lin and Schneeweiss 2016). The results obtained from RCTs are through precise controls and close monitoring of those specific participants, including diet control and exercise education. Although RCTs demonstrate the complete evaluation of drug efficacy and safety, they cannot completely reflect the real-world while patients in the real-world actually live in an uncontrolled environment and usually have a variety of comorbidities as well as inconsistent adherence to treatment (Park et al., 2021). As stated before, those reasons may have contributed to the relatively low weight loss extent in WM users compared with previous RCTs.

### Limitation

Our study had several limitations. First, it is hard to reflect on patients’ adherence to lifestyle modification and actual compliance between CHM users and WM users during the course of treatment in our retrospective observational study. Second, the prescription of the CHM may combine different formulas to achieve more holistic effects and it does not always exactly follow the consistent dosage or formula as well as WM. Third, since most subjects were Asian, the generalizability of these results for other ethnicities may be concerned. Forth, there were some baseline demographic differences between both groups. Despite the baseline differences in weight loss were adjusted in propensity score models, we still need further large randomized controlled trials to confirm the real causality of anti-obesity prescriptions and to collect comprehensive side effects.

## Conclusion

In conclusion, the results of our retrospective cohort study demonstrated that the CHM may provide a significant new dimension in our pursuit of weight control and prevention of metabolic syndrome by means of achieving body weight loss that was almost on a par with WM users in the real-world. Although the pharmacological mechanism of anti-obesity action is still vague and remains to be investigated, it is essential to conduct large-scale clinical trials and more longitudinal real-world studies for the potential of CHM in future in order to deliberate on the effectiveness and benefits of CHM in clinical practices along with assessing comorbidities and adverse events during follow-up. It is promising to develop a patient-centered long-term approach to weight management by providing the combined use of Western principles and Chinese medications therapeutic strategies.

## Data Availability

The original contributions presented in the study are included in the article/Supplementary Material, further inquiries can be directed to the corresponding author.
