# Corrigendum: Chinese herbal medicine is associated with higher body weight reduction than liraglutide among the obese population: a real-world comparative cohort study

**DOI:** 10.3389/fphar.2023.1222106

**Published:** 2023-05-23

**Authors:** Yu-Ning Liao, Hsing-Yu Chen, Ching-Wei Yang, Pai-Wei Lee, Chiu-Yi Hsu, Yu-Tung Huang, Tsung-Hsien Yang

**Affiliations:** ^1^ Division of Chinese Internal Medicine, Center for Traditional Chinese Medicine, Chang Gung Memorial Hospital, Taoyuan, Taiwan; ^2^ School of Traditional Chinese Medicine, College of Medicine, Chang Gung University, Taoyuan, Taiwan; ^3^ Graduate Institute of Clinical Medical Sciences, College of Medicine, Chang Gung University, Taoyuan, Taiwan; ^4^ Center for Big Data Analytics and Statistics, Chang Gung Memorial Hospital, Linkou Medical Center, Taoyuan, Taiwan; ^5^ Graduate Institute of Pharmacognosy, College of Pharmacy, Taipei Medical University, Taipei, Taiwan

**Keywords:** traditional Chinese medicine, obesity, overweight, weight loss, body mass index, liraglutide, weight control

In the published article, there was an error in the **Funding** statement for the Ministry of Science and Technology in Taiwan displayed as “grant No: MOST111-2320-B-182-009-MY3” instead of “grant No: MOST 111-2320-B-182-035-MY3”. The correct Funding statement appears below.

“Funding

This study was supported by Chang Gung Medical Foundation (grant No: CMRPG5I0011), the Ministry of Health and Welfare (M1107092) and the Ministry of Science and Technology in Taiwan (grant No: MOST 111-2320-B-182-035-MY3).”

The authors apologize for this error and state that this does not change the scientific conclusions of the article in any way. The original article has been updated.

