# Supplementary material for: Chinese herbal medicine is associated with higher body weight reduction than liraglutide among the obese population: A real-world comparative cohort study
Source: Front Pharmacol. 2022 Sep 9;13:978814. doi: 10.3389/fphar.2022.978814 (PMC9500198; doi:10.3389/fphar.2022.978814)
Supplement: Supplementary file 1 [file DataSheet1.docx]

Supplementary Material

| **Supplementary material table S1.** Sensitivity analysis for the changes in the primary end point of the CHM and WM users (use of IPTW) | | | | |
| --- | --- | --- | --- | --- |
|  | | | | |
| **With IPTW** | | **CHM users** | **WM users** | **p-value** |
| **Changes in Body Weight** | | | | |
|  | Kilograms of body weight | -4.45±4.79 | -2.04±4.79 | <0.001 |
|  | % of body weight | -5.39 | -2.45 | <0.001 |
|  | Loss of >5% body weight–% | 50.11 | 21.04 | <0.001 |
|  | Loss of >10% body weight–% | 16.74 | 5.1 | <0.001 |
|  | Changes in Body-Mass Index | -1.72±1.85 | -0.82±1.92 | <0.001 |
|  | Day  30 △ Body weight  60 △ Body weight  90 △ Body weight  120 △ Body weight  150 △ Body weight  180 △ Body weight | -3.04±3.46  -3.56±3.58  -4.2±3.9  -4.42±3.89  -4.56±4.08  -4.35±4.13 | -1.13±2.44  -1.47±3.14  -1.45±3.19  -1.6±3.36  -2.1±3.76  -1.96±3.87 | <.0001  <.0001  <.0001  <.0001  <.0001  <.0001 |

*The p-value was calculated by Pearson’s chi-squared test and Student’s t-test.

| **Supplementary material table S2.** Sensitivity analysis for the changes in the primary end point of the CHM and WM users (use of 1:1 PSM) | | | | |
| --- | --- | --- | --- | --- |
| **With 1:1 PSM** | | **CHM users**  (n=121) | **WM users**  (n=121) | **p-value** |
| **Changes in Body Weight** | | | | |
|  | Kilograms of body weight | -4.03±6.18 | -2.1±3.73 | 0.0036 |
|  | % of body weight | -4.77 | -2.51 | 0.0011 |
|  | Loss of >5% body weight–no. (%) | 49 (40.50) | 28 (22.95) | 0.0033 |
|  | Loss of >10% body weight–no. (%) | 18 (14.88) | 3 (2.46) | 0.0006 |
|  | Changes in Body-Mass Index | -1.56±2.41 | -0.91±1.87 | 0.0198 |
|  | Day  30 △ Body weight  60 △ Body weight  90 △ Body weight  120 △ Body weight  150 △ Body weight  180 △ Body weight | -2.78±4.49  -2.95±4.59  -3.39±4.53  -3.32±4.03  -3.57±3.88  -3.64±3.97 | -1.17±2.04  -1.34±2.41  -1.28±2.51  -1.4±2.88  -2.38±3.79  -2.09±3.75 | 0.0008  0.0017  <.0001  0.0002  0.0300  0.0020 |

*The p-value was calculated by Pearson’s chi-squared test and Student’s t-test.

**Supplementary material table S3.** Body weight changes among subgroups of different ages, comorbidities and BMI on treatment course Day30, 60, 90, 120, 150, and 180.

| A-1. subgroup analysis |  |  |  |
| --- | --- | --- | --- |
| **Day 30** | **CHM users** | **WM users** | **p-value** |
| △ Body weight | (n=701) | (n=659) |  |
| Age |  |  |  |
| age≧48 | -2.83±2.84 | -1.34±2.82 | <.0001 |
| age<48 | -3.04±4.39 | -1.16±2.45 | <.0001 |
| Hypertension |  |  |  |
| yes | -2.51±4.5 | -1.18±2.74 | 0.0114 |
| no | -3.02±3.83 | -1.44±2.67 | <.0001 |
| Diabetes mellitus |  |  |  |
| yes | -1.17±5.98 | -1.11±2.59 | 0.9582 |
| no | -3.06±3.75 | -1.86±3.03 | 0.0003 |
| BMI |  |  |  |
| 25≦BMI<30 | -2.46±2.59 | -0.94±2.41 | <.0001 |
| 30≦BMI<35 | -3.02±4.27 | -1.53±2.53 | <.0001 |
| 35≦BMI | -4.61±6.07 | -1.52±3.29 | <.0001 |

*The p-value was calculated by Pearson’s chi-squared test and Student’s t-test.

| A-2. subgroup analysis |  |  |  |
| --- | --- | --- | --- |
| **Day 60** | **CHM users** | **WM users** | **p-value** |
| △ Body weight | (n=701) | (n=659) |  |
| Age |  |  |  |
| age≧48 | -3.21±2.83 | -1.86±3.72 | <.0001 |
| age<48 | -3.63±3.74 | -1.29±2.89 | <.0001 |
| Hypertension |  |  |  |
| yes | -2.78±4.33 | -1.64±3.52 | 0.0322 |
| no | -3.57±3.31 | -1.69±3.36 | <.0001 |
| Diabetes mellitus |  |  |  |
| yes | -1.86±5.89 | -1.45±3.21 | 0.6756 |
| no | -3.57±3.24 | -2.42±4.17 | 0.0087 |
| BMI |  |  |  |
| 25≦BMI<30 | -3±2.71 | -1.19±3.15 | <.0001 |
| 30≦BMI<35 | -3.72±4.5 | -2.09±3.51 | 0.0002 |
| 35≦BMI | -4.72±3.36 | -1.74±3.71 | <.0001 |

*The p-value was calculated by Pearson’s chi-squared test and Student’s t-test.

| A-3. subgroup analysis |  |  |  |
| --- | --- | --- | --- |
| **Day 90** | **CHM users** | **WM users** | **p-value** |
| △ Body weight | (n=701) | (n=659) |  |
| Age |  |  |  |
| age≧48 | -3.7±3.51 | -1.87±3.47 | <.0001 |
| age<48 | -4.4±4.21 | -1.18±3.36 | <.0001 |
| Hypertension |  |  |  |
| yes | -3.85±4.19 | -1.43±3.34 | <.0001 |
| no | -4.26±3.92 | -1.95±3.59 | <.0001 |
| Diabetes mellitus |  |  |  |
| yes | -2.32±4.66 | -1.42±3.04 | 0.255 |
| no | -4.26±3.92 | -2.34±4.5 | <.0001 |
| BMI |  |  |  |
| 25≦BMI<30 | -3.64±3.02 | -1.13±2.59 | <.0001 |
| 30≦BMI<35 | -4.52±4.36 | -2.04±3.56 | <.0001 |
| 35≦BMI | -5.26±5.61 | -1.86±4.25 | <.0001 |

*The p-value was calculated by Pearson’s chi-squared test and Student’s t-test.

| A-4. subgroup analysis |  |  |  |
| --- | --- | --- | --- |
| **Day 120** | **CHM users** | **WM users** | **p-value** |
| △ Body weight | (n=701) | (n=659) |  |
| Age |  |  |  |
| age≧48 | -3.89±3.15 | -2.02±3.36 | <.0001 |
| age<48 | -4.7±4.34 | -2.01±7.22 | <.0001 |
| Hypertension |  |  |  |
| yes | -3.72±3.62 | -1.87±5.33 | 0.0005 |
| no | -4.51±4.02 | -2.29±4.02 | <.0001 |
| Diabetes mellitus |  |  |  |
| yes | -2.46±4.27 | -1.74±4.89 | 0.4025 |
| no | -4.53±3.94 | -3.03±4.82 | 0.0048 |
| BMI |  |  |  |
| 25≦BMI<30 | -3.85±3.19 | -1.77±5.74 | <.0001 |
| 30≦BMI<35 | -4.62±4.41 | -1.95±3.57 | <.0001 |
| 35≦BMI | -6.04±5.09 | -2.49±4.76 | <.0001 |

*The p-value was calculated by Pearson’s chi-squared test and Student’s t-test.

| A-5. subgroup analysis |  |  |  |
| --- | --- | --- | --- |
| **Day 150** | **CHM users** | **WM users** | **p-value** |
| △ Body weight | (n=701) | (n=659) |  |
| Age |  |  |  |
| age≧48 | -3.94±3.43 | -2.45±3.86 | <.0001 |
| age<48 | -4.95±4.44 | -2.39±6.6 | <.0001 |
| Hypertension |  |  |  |
| yes | -3.77±3.42 | -2.33±5.25 | 0.0038 |
| no | -4.7±4.21 | -2.58±4.58 | <.0001 |
| Diabetes mellitus |  |  |  |
| yes | -2.43±3.91 | -2.2±5.08 | 0.7847 |
| no | -4.72±4.12 | -3.3±4.62 | 0.0013 |
| BMI |  |  |  |
| 25≦BMI<30 | -3.95±3.3 | -2.19±5.68 | <.0001 |
| 30≦BMI<35 | -4.93±4.52 | -2.59±4.08 | <.0001 |
| 35≦BMI | -6.29±5.39 | -2.57±4.96 | <.0001 |

*The p-value was calculated by Pearson’s chi-squared test and Student’s t-test.

| A-6. subgroup analysis |  |  |  |
| --- | --- | --- | --- |
| **Day 180** | **CHM users** | **WM users** | **p-value** |
| △ Body weight | (n=701) | (n=659) |  |
| Age |  |  |  |
| age≧48 | -3.83±3.69 | -2.37±3.77 | <.0001 |
| age<48 | -4.96±4.85 | -2.14±7.85 | <.0001 |
| Hypertension |  |  |  |
| yes | -3.76±3.46 | -1.93±3.62 | <.0001 |
| no | -4.68±4.62 | -2.89±7.55 | 0.0005 |
| Diabetes mellitus |  |  |  |
| yes | -2.51±3.19 | -2.17±5.65 | 0.5456 |
| no | -4.69±4.55 | -2.74±4.86 | <.0001 |
| BMI |  |  |  |
| 25≦BMI<30 | -3.82±3.59 | -1.68±3.06 | <.0001 |
| 30≦BMI<35 | -4.98±4.12 | -2.32±4.05 | <.0001 |
| 35≦BMI | -6.45±6.81 | -3.21±8.77 | 0.0005 |

*The p-value was calculated by Pearson’s chi-squared test and Student’s t-test.

**Supplementary material table S4.** Diagnosis codes used in the study.

| Disease Name | ICD-9-CM codes | ICD-10 codes |
| --- | --- | --- |
| Hypertension | 4010, 4011, 4019 | I10 |
| Dyslipidemia | 2722, 2724, 2729 | E782, E784, E785 |
| Ischaemic heart diseases | 4111, 4130, 4131, 4139, 410, 4295, 4296, 42971, 42979, 4110, 41181, 41189, 412, 414 | I20, I201, I208, I209, I21, I211, I212, I213, I214, I219, I22, I221, I228, I229, I23, I231, I232, I233, I234, I235, I236, I238, I24, I241, I248, I249, I25, I251, I252, I255, I256, I258, I259 |
| CVD | 36234, 430, 431, 432, 433, 434, 435, 436, 437, 438 | G45, G46, H340, I60, I61, I62, I63, I64, I65, I66, I67, I68, I69 |
| MI | 410, 412 | I21, I22, 252 |
| CHF | 39891, 40201, 40211, 40291, 40401, 40403, 40411, 40413, 40491, 40493, 4254, 4255, 4256, 4257, 4258, 4259, 428 | I099, I110, I130, I132, I255, I420, I425, I426, I427, I428, I429, I43, I50, P290 |
| PVD | 0930, 4373, 440, 441, 4431, 4432, 4433, 4434, 4435, 4436, 4437, 4438, 4439, 5571, 5579 | 0930, 4373, 440, 441, 4431, 4432, 4433, 4434, 4435, 4436, 4437, 4438, 4439, 5571, 5579 |
| Dementia | 290, 2941, 3312 | F00, F01, F02, F03, F051, G30, G311 |
| Rheumatic | 4465, 7100, 7101, 7102, 7103, 7104, 7140, 7141, 7142, 7148, 725 | M05, M06, M315, M32, M33, M34, M351, M353, M360 |
| Ulcer | 531, 532, 533, 534 | K25, K26, K27, K28 |
| Hemiplegia | 3341, 342, 343, 3440, 3441, 3442, 3443, 3444, 3445, 3446, 3449 | G041, G114, G801, G802, G81, G82, G830, G831, G832, G833, G834, G839 |
| Renal disease | 40301, 40311, 40391, 40402, 40403, 40412, 40413, 40492, 40493, 582, 5830, 5831, 5832, 5833, 5834, 5835, 5836, 5837, 585, 586, 5880 | V420, V451, V56, I120, I131, N032, N033, N034, N035, N036, N037, N052, N053, N054, N055, N056, N057, N18, N19, N250, Z490, Z491, Z492, Z940, Z992 |
| MSLD | 4560, 4561, 4562, 5722, 5723, 5724, 5725, 5726, 5727, 5728 | I850, I859, I864, I982, K704, K711, K721, K729, K765, K766, K767 |
| CPD | 4168, 4169, 490, 491, 492, 493, 494, 495, 496, 500, 501, 502, 503, 504, 505, 5064, 5081, 5088 | I278, I279, J40, J41, J42, J43, J44, J45, J46, J47, J60, J61, J62, J63, J64, J65, J66, J67, J684, J701, J703 |
| MLD | 07022, 7023, 7032, 7033, 7044, 7054, 706, 709, 570, 571, 5733 5734, 5738, 5739 | V427, B18, K700, K701, K702, K703, K709, K713, K714, K715, K717, K73, K74, K760, K762, K763, K764, K768, K769, Z944 |
| DM with chronic disease | 2504, 2505, 2506, 2507 | E102, E103, E104, E105, E107, E112, E113, E114, E115, E117, E122, E123, E124, E125, E127, E132, E133, E134, E135, E137, E142, E143, E144, E145, E147 |
| DM without chronic disease | 2500, 2501, 2502, 2503, 2508, 2509 | E100, E101, E106, E108, E109, E110, E111, E116, E118, E119, E120, E121, E126, E128, E129, E130, E131, E136, E138, E139, E140, E141, E146, E148, E149 |
| Malignancy | 140, 141, 142, 143, 144, 145, 146, 147, 148, 149, 150, 151, 152, 153, 154, 155, 156, 157, 158, 159, 160, 161, 162, 163, 164, 165 , 166, 167, 168, 169, 170, 171, 172, 174, 175, 176, 177, 178, 179, 180, 181, 182, 183, 184, 185, 186, 187, 188, 189, 190, 191, 192, 193, 194, 1950, 1951, 1952, 1953, 1954, 1955, 1956, 1957, 1958, 200, 201, 202, 203, 204, 205, 206, 207, 208, 2386 | C00, C01, C02, C03, C04, C05, C06, C07, C08, C09, C10, C11, C12, C13, C14, C15, C16, C17, C18, C19, C20, C21, C22, C23, C24, C25, C26, C30, C31, C32, C33, C34, C37, C38, C39, C40, C41, C43, C45, C46, C47, C48, C49, C50, C51, C52, C53, C54, C55, C56, C57, C58, C60, C61, C62, C63, C64, C65, C66, C67, C68, C69, C70, C71, C72, C73, C74, C75, C76, C81, C82, C83, C84, C85, C88, C90, C91, C92, C93, C94, C95, C96, C97 |
| Cancer | 196, 197, 198, 199 | C77, C78, C79, C80 |
| AIDS | 042, 043, 044 | B20, B21, B22, B24 |
| Chronic hepatitis | 57149, 57140 | K738, K739 |
| Fatty liver | 5718 | K760 |
| Nonalcoholic steatohepatitis | 5718, 5733 | K7581 |
| Cerebrovascular disease | 430, 431, 432 | I60, I601, I602, I603, I604, I605, I606, I607, I608, I609, I61, I611, I612, I613, I614, I615, I616, I618, I619, I62, I621, I629 |
| Brain disease | 433, 434 | I63, I631, I632, I633, I634, I635, I636, I638, I639, I64, I65, I651, I652, I653, I658, I659, I66, I661, I662, I663, I664, I668, I669 |
| Hypertensive encephalopathy | 4372 | I674 |

*Abbreviations: ICD-9-CM, International Classification of Disease, 9^th^ Revision, Clinical Modification; ICD-10, International Classification of Disease, 10^th^ Revision.
